# Abdominal cocoon with bilateral cryptorchidism and seminoma in the right testis: a case report and review of literature

**DOI:** 10.1186/s12893-019-0636-z

**Published:** 2019-11-11

**Authors:** Bingqing Yue, Zilian Cui, Weiting Kang, Hanbo Wang, Yuzhu Xiang, Zhilong Huang, Xunbo Jin

**Affiliations:** 1 Minimally Invasive Urology Center, Shandong Provincial Hospital affiliated to Shandong University, Jinan, Shandong China; 2 Shandong University School of Medicine, Jinan, Shandong China

**Keywords:** Abdominal cocoon, Cryptorchidism, Seminoma, Clinical manifestation, Diagnosis, Treatment

## Abstract

**Background:**

Abdominal cocoon is a rare peritoneal lesion and is difficult to diagnose because of its lack of special clinical manifestations. Until now, there is no case report of abdominal cocoon combined with cryptorchidism and seminoma.

**Case presentation:**

A case of abdominal cocoon with cryptorchidism and seminoma was diagnosed and treated in our hospital. The patient had no symptoms except occasional abdominal pain. He underwent laparoscopy because of bilateral cryptorchidism and seminoma in the right testis. During the surgery, he was diagnosed with abdominal cocoon due to the thick fibrous tissues which was tightly adhered and encased part of intestine like a cocoon. Enterolysis and bilateral cryptochiectomy were performed after the diagnosis and nutritional and symptomatic support was provided after the surgery. The patient recovered well and was discharged soon. The postoperative pathological examination confirmed the presence of bilateral cryptorchidism and seminoma in the patient’s right testis.

**Conclusion:**

There are only a handful of cases where a patient has both abdominal cocoon and cryptorchidism. Since the etiologies of both diseases remain unknown, further research is required to investigate effective diagnosis and treatment for the diseases and explore the potential connection between the two diseases.

## Background

Abdominal cocoon is a rare peritoneal lesion characterized by a layer of dense and pale abnormal fibro-collagenous membrane that encases part or all of intestine like a cocoon. The most common symptoms of the disease are abdominal pain, abdominal distension, vomiting, nausea, and abdominal mass, all of which are, however, not specific to abdominal cocoon. The first case of abdominal cocoon was reported by Owtschinnikow in 1907 when he referred the disease as ‘peritonitis chronica fibrosa incapsulata’ [1] while the name ‘abdominal cocoon’ was firstly used by Foo et al. in 1978 [2]. Cryptorchidism is a common male genital tract malformation in newborns characterized by the remaining of one or both of the testes in an abnormal position during the testis descending process from the retroperitoneum into the scrotum. Cryptorchidism is prone to malignant changes, and seminoma is the most common testicular malignant tumor. Cases of abdominal cocoon combined with cryptorchidism are extremely rare with only 2 reported cases [3, 4]. To the best of our knowledge, no case of abdominal cocoon with cryptorchidism and seminoma has been reported to date. Ethics approval of this study was obtained from the Ethical Committee of the Shandong Provincial Hospital affiliated to Shandong University. Written informed consent was obtained from the patient for publication of this case report and any accompanying images.

## Case presentation

A 28-year-old male patient was admitted to the Minimally Invasive Urology Center of Shandong Provincial Hospital. The patient complained of bilateral scrotal vacuity which had been bothered him since his childhood. Two years ago when he was diagnosed with cryptorchid nodules in his right testis. He has been constantly bothered by symptoms such as occasional abdominal pain, nausea, and vomiting for 10 years. He had a history of syphilis for 2 years, which had been treated with penicillin. Laboratory examinations including the routine blood test, liver function tests, coagulation analysis, urinalysis, and tumor biomarker tests such as alpha fetoprotein(AFP), carcinoembryonic antigen(CEA) and Human Chorionic Gonadotropin(HCG) were showing no abnormality. His syphilis was confirmed in the present study by a positive gelatin agglutination test result and the result of rapid plasma reactivity (1:2). The sex hormone assay demonstrated an increase of follicular stimulating hormone (FSH, 67.1 mIU/ml), luteinizing hormone (LH, 20.56 mIU/ml), and prolactin (P, 18.93 ng/ml) and a decrease of the estrogen (E, 17.04 pg/ml). However, the testosterone(T) was confirmed to be at a normal level (5.23 ng/ml).

**Fig. 1:**
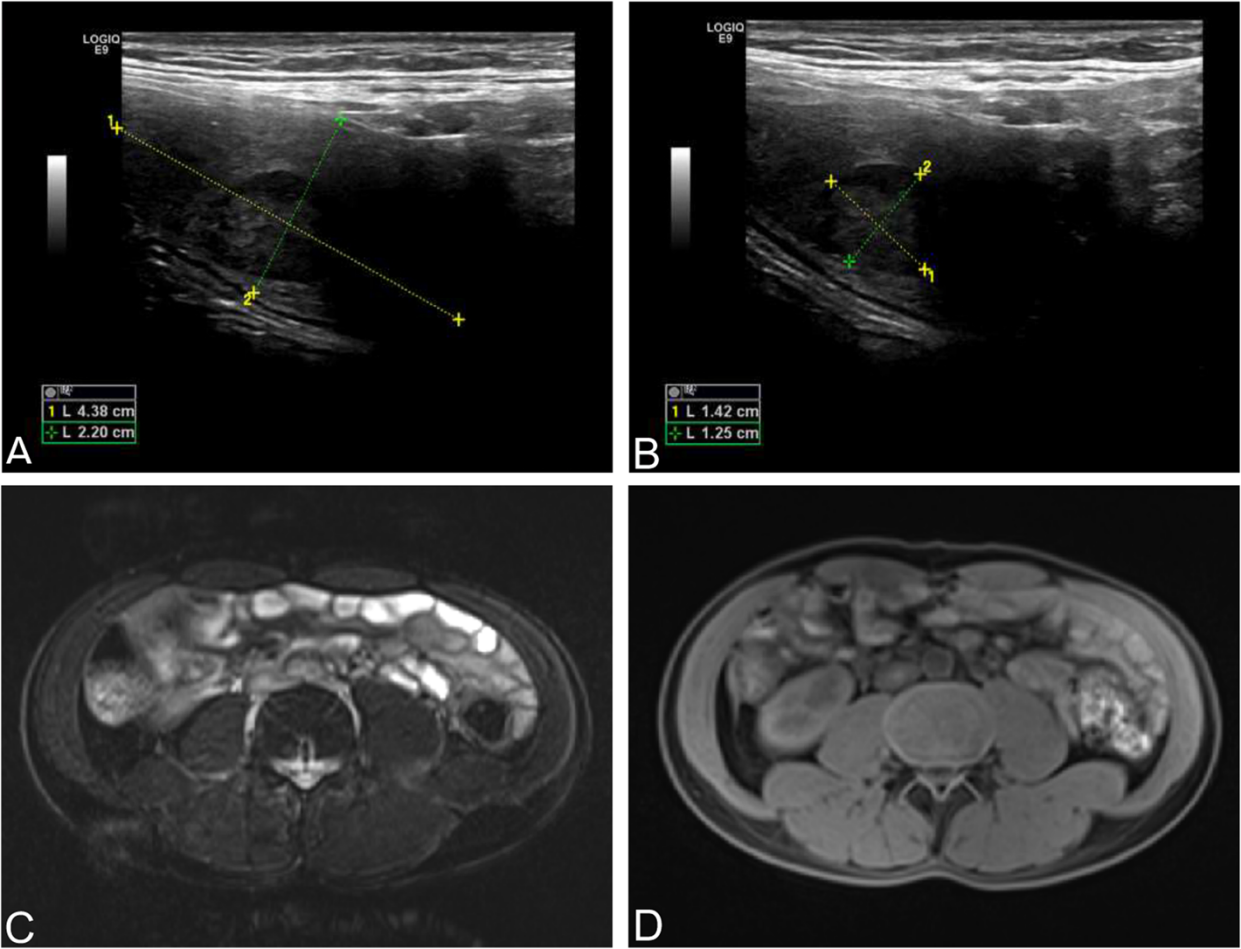
Imageological examination. **a**, **b** Abdominal ultrasonography showed the right undescended testicle in the abdomen, whose size was about 4.4 × 2.2 cm (**a**). Two regular and well-defined hypoechoic nodules were found in the right testicle with the larger one of about 1.42 × 1.25 cm (**b**). **c**, **d** The images of abdominal Magnetic Resonance Imaging (MRI)

The ultrasonography showed bilateral cryptorchidism and cryptorchid nodules (Fig. 1a and b). The abdominal Magnetic Resonance Imaging (MRI) revealed bilateral scrotal vacuity without clear testicular structure. In the anterior inner side of the right iliac vessel, a long and well-defined T1 and T2 signal (displayed as a shade) with a size of approximately 2.4 × 2.8 cm was identified as a suspected ectopic testicle. On the left side, a signal shade of approximately 0.7 × 1.3 cm was detected in the corresponding area. Both kidneys were of regular size and shape. A long circular T1 and T2 signal (signifying a lesion) with a diameter of about 0.4 cm was found in the lower posterior portion of the left kidney. No abnormalities were discovered in the remaining organs (Fig. 1c and d).

**Fig. 2:**
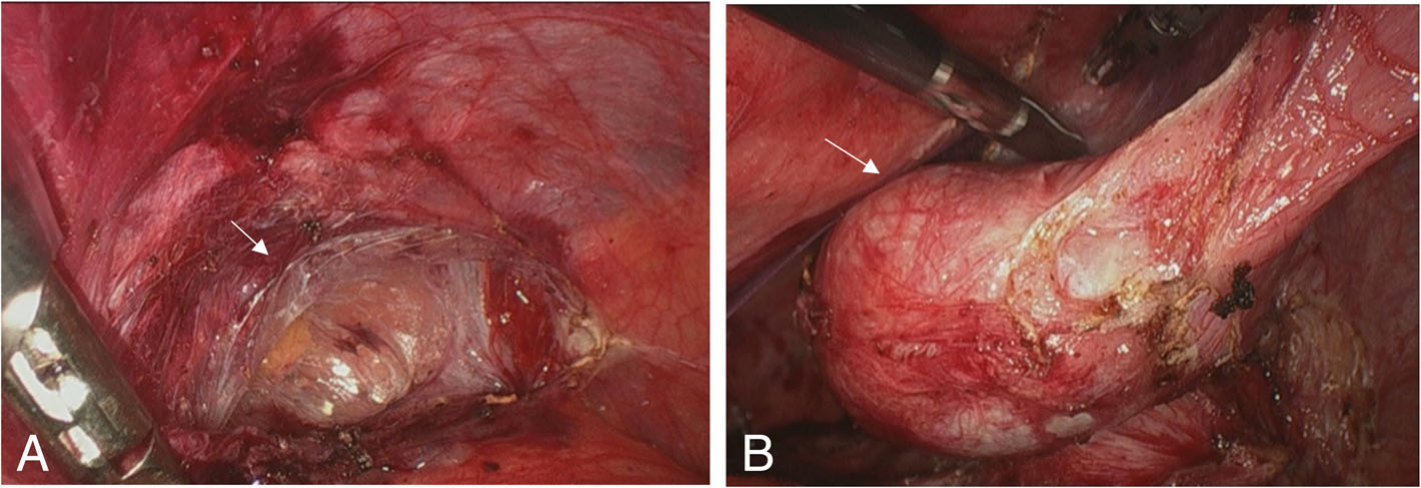
Intraoperative images. Severe abdominal adhesions are detected (**a**) and the cryptorchidism was surrounded by fibrous tissues (**b**)

**Fig. 3:**
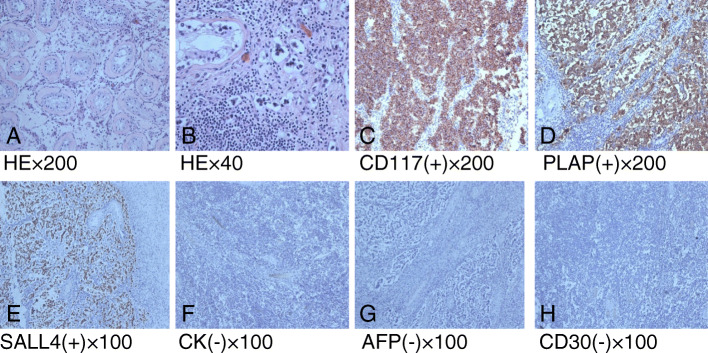
Pathologic examination. **a** Cryptorchidism. (HE× 200) The basement membrane of spermatogenic tubule thickened with hyaline degeneration. The number of support cells and interstitial Leydig cells increased, and no spermatocyte was found in the testes. **b** Seminoma. (HE× 400) The number of heteromorphic cells and interstitial lymphocyte increased. **c**-**e**: CD117(**c**), PLAP(**d**), and SALL4(**e**) were shown to be positive in the tumor cells by the immunohistochemical staining. **f**-**h** CK(**f**), AFP(**g**) and CD30(**h**) were shown to be negative in the tumor cells by the immunohistochemical staining

Laparoscopy was performed in our hospital in August 2018 to proceed with further diagnosis. During the operation, we found adhesions between the patient’s bowel loops and peritoneum. In addition, fibrous tissues were detected between small intestines, which fixed the intestines and made it hard to separate them (Fig. 2). The right cryptorchid testis was found above the inner inguinal ring while the left cryptorchid testis was identified near the proximal end of the left ductus deferens. The patient underwent enterolysis and bilateral cryptorchiectomy. Postoperative pathological examination revealed the thickening of spermatogenic tubule basement membrane in the left testicular tissues with hyaline degeneration. The increase in the number of supporting cells and Leydig cells and the absence of spermatogenic cells demonstrated the pathological changes of cryptorchidism in the right testis. The mass in the right cryptorchid testis was confirmed as a seminoma with interstitial infiltration. There was no tumor identified in epididymis and vas deferens (Fig. 3).

## Discussion and conclusion

Abdominal cocoon is also known as primary sclerosing peritonitis, idiopathic sclerosing peritonitis or sclerosing encapsulating peritonitis [5]. Medicine intake, abdominal surgery, organ transplantation, tuberculosis bacillus infection, inflammatory disorders, peritoneal dialysis are all risk factors for secondary abdominal cocoon [6]. The cause of the idiopathic abdominal cocoon is still unclear, which may be related to congenital fetal dysplasia. Cryptorchidism refers to the failure of the testicle in passing down into the scrotum, which results in the absence of one or both testes from the scrotum. It can be divided into congenital cryptorchidism and acquired cryptorchidism [7]. At present, the etiology and mechanism of undescended testicles in the embryonic development are also yet to be explored. For patients with abdominal cocoon combined with cryptorchidism, it is possible that the fibrosis formed during the development of embryos causes the extensive abdominal adhesion, which further affects the testicular descending process.

So far, the preoperative diagnosis of abdominal cocoon is still difficult, and most patients are diagnosed by finding fibrous capsule shaped like silkworm chrysalis during laparotomy exploration [8]. The lack of specificity in the early clinical manifestations of abdominal cocoon has been an impediment to the accurate and prompt diagnosis of the disease. Some patients have no symptoms at all, but only accidentally detected abdominal cramps during the surgical exploration of other diseases. Some patients may complain of intermittent abdominal pain, abdominal distension, nausea, vomiting, anorexia and malnutrition, and even complete or incomplete ileus [9].

Abdominal X-ray may show dilated bowels with air-fluid levels in a case of abdominal cocoon. A Barium meal can demonstrate that the bowels gather in the center of the abdomen in the shape of a large cauliflower. Ultrasonography can be employed to reveal the dilated intestinal loops surrounded by a thick hypoechoic fibrous membrane [5]. Computed tomography(CT) is the most useful imaging technique in the diagnosis of abdominal cocoon at the moment [10]. The common manifestation of the disease is that the small intestine is partially or completely encased by a fibrous membrane [11]. The adhesion and fixation between the small intestines lead to the expansion of adjacent intestines. Other imaging features of the disease include a small amount of ascites, peritoneal thickening or enhancement, and localized or diffuse peritoneal calcification [12]. Magnetic Resonance Imaging (MRI), a medical imaging technique used in radiology to form pictures, is a better way to detect bowel adhesions and peritoneum thickening caused by abdominal cocoon [10]. Histopathologically, the hypoechoic fibrous membrane of a patient with abdominal cocoon is usually fibrous tissues with or without local inflammatory reaction.

Cryptorchidism can be diagnosed by physical examinations and ultrasonography. Currently, laparoscopy is the gold standard for the diagnosis of cryptorchidism. In the present study, the imaging examination did not find the exact position of the left cryptorchid testis in the abdominal cavity nor obvious imaging characteristics of abdominal cocoon. However, during the laparoscopic exploration, we detected severe abdominal cavity adhesions and fixed intestinal loops gathered which were attached to fibrous tissues. The left ectopic testis was identified during the operation. The patient had no diseases other than syphilis. As he was born with bilateral scrotal vacuity, the diagnosis was idiopathic abdominal cocoon complicated with bilateral congenital cryptorchidism and seminoma in the right testis.

The treatment of abdominal cocoon includes conservative treatment and surgical treatment. Patients with mild symptoms can receive conservative treatments such as nutritional support, gastrointestinal decompression, and medications. Corticosteroids, colchicine, immunosuppressive agents (such as MMF, azathioprine, cyclosporine, etc.) can inhibit the inflammatory response, and tamoxifen has antifibrotic effects, all of which can be employed to treat abdominal cocoon [13]. Surgical treatment such as enterolysis is recommended for patients who have a long history of recurrent intestinal obstruction and demonstrate no effective recovery through medication. Enterectomy is performed for patients with enteric necrosis. Surgery can relieve adhesions and obstruction, and effectively reduce the incidence of postoperative re-obstruction. However, there are rare but probable cases when surgical complications such as intestinal fistula, short bowel syndrome, and intestinal dysfunction occur during the surgical treatment [14]. It has also been reported that the postoperative combination of steroids or tamoxifen can help reduce the risk of recurrence [15].

Treatment of adult cryptorchidism includes cryptorchidectomy and orchidopexy. As the incidence of malignant tumors in adult cryptorchid testes is extremely high, cryptorchidectomy is the most recommended treatment. There are cases when a patient chooses orchidopexy to retain the function of secreting sex hormones. Preoperative and postoperative adjuvant hormone therapy may reduce surgical difficulties and improve fertility in patients with undescended testes. Spinelli et al. reported that testicular volume increased significantly after 5 years of follow-up in patients with a normalized testicular atrophy index (TAIn) > 20% receiving gonadotropin releasing hormone analog (GnRHa) before and after surgery [16]. Besides, moving an ectopic testis to its proper location in the scrotum can facilitate the timely detection of malignant transformation in testes. However, in the cases of cryptorchidism with malignant tumors, surgical resection should be performed as soon as possible. In the present study, the preoperative ultrasonography revealed a nodule in the right cryptorchid testis which may be a malignant tumor. As such, cryptorchidectomy was performed, and the postoperative pathological examination confirmed seminoma in the right testis.

Testicular seminoma is the most common malignant tumor in testes which has low malignancy and grows slowly. There are three clinical stages of testicular seminoma:I stage is limited to the testicles with no lymph node metastasis; II stage includes regional lymph node metastasis; III stage has distant lymph node metastasis or lung metastasis.

Of a single morphological structure and a uniform size, classic seminoma cells are often arranged in the form of nests or sheets and separated by fibrous tissues with a large number of infiltrating lymphocytes. From the perspective of immunohistochemistry, most seminoma could express placental alkaline phosphatase (PLAP) tumor marker, especially in seminoma and ovarian cancer. It has also been verified by Kemmer et al. that 82% of seminoma can express CD117 [17]. Immunohistochemistry examinations of seminoma can also show the negative result of CD30, AFP, and CK, with which we can differentiate seminoma from embryonal carcinomas, yolk sac tumors, and choriocarcinomas.

Radical orchiectomy is generally recommended for testicular tumors. Approximately 80 to 85% of patients with seminoma of clinical stage I can be cured by the surgery [18]. Standard postoperative adjuvant regimens include surveillance, radiotherapy, and carboplatin-based chemotherapy. Surveillance is suitable for patients with good compliance while adjuvant radiotherapy or chemotherapy should be offered to patients who could not be regularly monitored. At present, the radiotherapy recommended by NCCN guidelines is 20 Gy in 10 fractions to para-aortic fields [19]. Chemotherapy has been used as an alternative therapy in recent years due to the carcinogenic and cardiotoxic effects of radiation therapy. Studies have found that postoperative single-dose of carboplatin (400 mg/m2 or an area under the concentration-time curve of 7) can reduce the recurrence rates to 1.8%~ 8.6% [20].

In the present study, the testicular seminoma with which the patient was diagnosed was identified as clinical stage I since no tumors were found in other parts of his body in the preoperative examination. As the patient suffered from intestinal damage during the operation, he was given a diet ban for 2 weeks to prevent intestinal fistula, while receiving parenteral nutrition and other symptomatically supportive treatment at the same time. The patient recovered well and was discharged 23 days after the surgery. During the follow-up, no tumor recurrence. The patient’s intestinal obstruction recurred 6 months after the operation, and improved after conservative treatment.

Even though there are only a few cases of idiopathic abdominal cocoon complicated with cryptorchidism, it is still worth exploring since there is a possible connection between the two diseases both of which are related to the abnormal embryonic development. A sensible assumption could be that the intestine loops enclosed by the dense fibrous membrane may be responsible for keeping the cryptorchid testis from descending into the scrotum. However, a precise determination of their relationship is still yet to be made and thus calls for further research in this regard. Patients diagnosed with cryptorchidism should be alert to the possibility of abdominal cocoon in order to avoid missed diagnosis and misdiagnosis and reduce the incidence of surgical complications.

## Data Availability

The datasets used during the current study are available from the corresponding author on reasonable request.
